# Evaluating capacity at three government referral hospital emergency units in the kingdom of Eswatini using the WHO Hospital Emergency Unit Assessment Tool

**DOI:** 10.1186/s12873-020-00327-w

**Published:** 2020-05-06

**Authors:** J. L. Pigoga, A. P. Joiner, P. Chowa, J. Luong, M. Mhlanga, T. A. Reynolds, L. A. Wallis

**Affiliations:** 1 Division of Emergency Medicine, University of Cape Town, Anzio Road, Observatory, Cape Town, 7935 South Africa; 2 Division of Emergency Medicine, Duke University, Durham, North Carolina USA; 3 Department of Emergency Medicine, Emory University, Atlanta, GA USA; 4 Department of Emergency Medicine, The Ohio State University, Columbus, OH USA; 5 Emergency Preparedness and Response, Eswatini Ministry of Health, Mbabane, Eswatini; 6 Department for Management of NCDs, Disability, Violence and Injury Prevention, World Health Organization, Geneva, Switzerland

**Keywords:** Emergency medicine, Emergency care, Health systems development, Global health

## Abstract

**Background:**

The Kingdom of Eswatini, a lower-middle income nation of 1.45 million in southern Africa, has recently identified emergency care as a key strategy to respond to the national disease burden. We aimed to evaluate the current capacity of hospital emergency care areas using the WHO Hospital Emergency Unit Assessment Tool (HEAT) at government referral hospitals in Eswatini.

**Methods:**

We conducted a cross-sectional study of three government referral hospital emergency care areas using HEAT in May 2018. This standardised tool assists healthcare facilities to assess the emergency care delivery capacity in facilities and support in identifying gaps and targeting interventions to strengthen care delivery within emergency care areas. Senior-level emergency care area employees, including senior medical officers and nurse matrons, were interviewed using the HEAT.

**Results:**

All sites provided some level of emergency care 24 h a day, 7 days a week, though most had multiple entry points for emergency care. Only one facility had a dedicated area for receiving emergencies and a dedicated resuscitation area; two had triage areas. Facilities had limited capacity to perform signal functions (life-saving procedures that require both skills and resources). Commonly reported barriers included training deficits and lack of access to supplies, medications, and equipment. Sites also lacked formal clinical management and process protocols (such as triage and clinical protocols).

**Conclusions:**

The HEAT highlighted strengths and weaknesses of emergency care delivery within hospitals in Eswatini and identified specific causes of these system and service gaps. In order to improve emergency care outcomes, multiple interventions are needed, including training opportunities, improvement in supply chains, and implementation of clinical and process protocols for emergency care areas. We hope that these findings will allow hospital administrators and planners to develop effective change management plans.

## Background

The majority of deaths related to emergency conditions occur in low- and middle-income countries (LMICs) [1]. The African continent in particular carries a disproportionate burden of these emergencies and their related mortality, and clinical outcomes are also worse in the region compared to high-income countries [2]. Emergencies encompass a range of health conditions requiring timely recognition and treatment, including medical, traumatic and surgical, and obstetric aetiologies. Challenges associated with building a robust system to manage such cases are intensified in limited-resource settings [3]. In spite of these difficulties, improving emergency care systems in these settings is of paramount importance, as doing so is likely to reduce death and disability substantially [2].

The Kingdom of Eswatini – formerly known as the Kingdom of Swaziland - is a small, landlocked southern African nation covering approximately 17,000 km^2^, with a population of 1.45 million and a per capita income of 9800 USD [4] [5] [6]. Like many of its regional neighbours, Eswatini faces a high burden of emergency health conditions. Despite an increasing gross domestic product and expenditure of nearly 10% of its national budget on healthcare [5] [7], health outcomes remain poor [6]. Emergency care has been shown to be cost- and time-effective, and timely emergency care has been linked to better outcomes from many of the conditions that burden the nation, including acute respiratory infections, diarrhoeal disease, injuries, and obstetric emergencies [ 8]. Given this, the Kingdom of Eswatini Ministry of Health (MoH) has identified emergency care strengthening as a key mechanism to improve health outcomes and move the country towards universal health coverage.

Although there is an emphasis on prehospital emergency care, formalisation of facility-based emergency care provision has been slower. Many rural hospitals and clinics lack dedicated emergency units: instead, most facilities accept patients presenting with emergencies alongside other non-emergent patients [2] [9]. Emergency medicine, the specialty that oversees both facility-based and prehospital care for acute injury and illness, is not recognised and there are no specialist emergency physicians or dedicated training programmes in-country [10]. As a result, staff almost always lack formal training in the management of medical and traumatic emergencies and may not be able to effectively prioritise emergency patients among all those who present to their departments. While emergency care was assessed previously in 2014, the results were limited [10]. There is a need for more specifics in order to develop a larger improvement strategy. At the request of the MoH, to support the government health system strengthening agenda, we undertook an assessment of emergency care capacity at referral level hospitals’ emergency care areas in Eswatini using the WHO Hospital Emergency Unit Assessment Tool (HEAT).

## Methods

A cross-sectional study of three government referral hospital using the HEAT was undertaken in May 2018. HEAT is a standardised tool assists healthcare facilities to assess the emergency care delivery capacity in facilities, and support in identifying gaps and targeting interventions to strengthen emergency care delivery. HEAT findings can bolster emergency care not only within a specific facility, but also in the healthcare system more generally.

This study evaluated government hospitals in Eswatini that receive referrals; there are four regional facilities – all in rural environments - and one tertiary facility in the nation’s capital of Mbabane. Due to limited time and resources, two of the country’s four regional hospitals were selected for inclusion using a simple random number generator. The single tertiary site was also targeted for this study. Selected study sites were notified by MoH officials via telephone and letter regarding the study and visit dates.

Researchers worked in collaboration with MoH personnel to administer the tool during a one-day site visit. Senior-level emergency care area employees were selected by the Head of each hospital to participate: each site identified a senior medical officer, a nurse matron, and another clinical officer or nurse within the department. Patients were not involved in design, or conduct, or reporting, or dissemination plans of our research. Written informed consent was obtained in English prior to participation. A verbal summary of the study was provided by researchers, after which participants were given time to read the consent form and ask any questions. Local translation was provided by MoH personnel where necessary to ensure complete understanding of the form.

Researchers were trained and followed a standardised procedure for collecting data in individual meetings with key informants. Both the primary researcher and participant had a printed copy of the tool. The primary researcher conducted an interview on each section while the secondary researcher recorded each answer on a form.

The HEAT assesses facility infrastructure, human resources, availability of clinical services (including opening hours), and clinical guidance such as protocols and checklists. The final section evaluates “signal functions” (a simple mechanism for identifying the presence of both the skills and resources needed to perform life-saving procedures), assessing the unit’s capacity to deliver core, high-impact emergency care interventions for potentially life-threatening conditions, including respiratory distress, shock, sepsis, altered mental status, complications of pregnancy, and serious injury [ 11]. The ability to execute these key actions serves as an overall marker of emergency care capacity [12]. In conjunction with information gained from the other sections, signal functions allow for rapid assessment of emergency care delivery: any limited availability of these key interventions signals a critical gap in delivery capacity for which a cause should be identified so that targeted improvements can be made.

HEAT uses four types of questions to evaluate emergency care capacity:

1. Open-ended objective (e.g., name of facility);
2. Number response (e.g., number of emergency unit visits per year);
3. Discrete answers (e.g., yes or no); and
4. Availability rating.

The availability rating questions are used to assess resource and service capacities, specifically the ability to perform signal functions in the time frame needed for emergency care. These questions are meant to reflect the demand-side factors (e.g., number of patients in need) for the service, as well as the supply-side factors (e.g., sufficient resources, satisfactory training). For each of these questions, the resource or service is noted as: 1 - generally unavailable; 2 - somewhat available (available to only some of those who need it); or 3 - adequate (present and available to almost everyone in need and used when needed).

Wherever availability ratings were less than adequate (below 3), the factors that contribute to its deficiency were explored. Responses were coded as below (Table 1).

**Table 1:** Barriers to availability of critical HEAT resources, services, and functions

Barrier	Description
Infrastructure	Inadequate physical space, electricity, or water
Absent equipment	The resource was not present at the facility
Broken equipment	The resource was present, but not in working order
Stock out	The resource, service or function could not be procured, or required supplies out of stock often due to stock management practices or procurement failures (e.g., reagents, tubes, IV catheters)
Training	Staff knowledge/skill gaps limited capacity to use the resource, provide a service, or perform a function
Personnel	The resource, service or function was available, but lack of adequate numbers of staff limited capacity
User fees	The resource, service or function was available, but an out-of-pocket payment requirement prevented care delivery
Opening hours	Hours the facility can be accessed by acute patients
Other	Other barrier(s) not listed above, for which explanation(s) were recorded verbatim
Unknown	Participant was not able to identify rationale behind inadequate resource, service or function

HEAT completion times averaged approximately 1 h. Results for participants at each site were collated. Multiple answers were dealt with by taking averages or the majority answer, where appropriate. Data were entered into encrypted Microsoft Excel (© Microsoft, Richmond, WA) spreadsheets, where basic descriptive statistics were generated. Study sites were provided with reports detailing both strengths and areas for potential improvement.

Ethical approval for this study was obtained from the Emory University Institutional Review Board and the Eswatini National Health Research Review Board.

## Results

A total of 11 key informants provided information across three facilities.

### Facility characteristics

The tertiary hospital served the nation’s entire population, while each regional hospital had a smaller catchment population of approximately 250,000. All hospitals reported capacity to provide emergency care 24 h per day. Hospitals were staffed by providers who were in-unit during opening hours; nurses remained in units overnight while higher-level providers were on-call for emergencies but off-campus. Two hospitals reported that their emergency care areas were staffed only with rotating providers assigned for periods of approximately 1 month, and did not have staff permanently assigned to emergency care areas. All sites reported having at least one operating theatre that was always available for emergency operations (Table 2).

**Table 2:** Facility metrics at referral hospitals in Eswatini

**Metric**	**Tertiary Hospital**	**Regional Hospital 1**	**Regional Hospital 2**
Inpatient beds	500	100	175
Inpatient admissions (per year)	5000	3500	6200
Operating theatres available 24 h a day, 7 days a week	1	2	1
Emergency operations (per year)	600	1300	800
Emergency visits (per year)	37,000	19,500	75,000
Patients arriving by ambulance	20%	17.5%	20%

All hospitals maintained two distinct areas for the delivery of emergency care. One was a casualty area for the treatment of injuries and the other an emergency area within the general outpatient department (OPD). Casualty and OPD areas were physically separated by some distance across campuses at two hospitals. In combination, these areas were able to provide 24-h emergency care services at each facility. For the purposes of responses reported below, we report on whether services could be performed in either of the above mentioned emergency care areas.

Two facilities had dedicated triage areas (Additional file 1). The tertiary hospital was the only facility with a dedicated resuscitation area. All facilities noted challenges with obtaining medications; two receive key medications from pharmacy when needed. Lack of equipment was noted in all facilities.

All hospitals had general laboratories with multiple diagnostic laboratory tests available, including full blood count and glucose testing. However, many tests, including rapid HIV testing, were generally unavailable due to reagent stockouts, and reporting of results not timely. Blood banks were located in all three hospitals.

### Human resources

All facilities reported receiving a wide range of medical and surgical cases (Additional file 2). Training deficits were noted by al hospitals; these included training related to critical trauma and airway interventions, and neonatal care. At all facilities, patients presenting with obstetric and gynaecological (OB/Gyn) complaints were ultimately sent to the maternity ward. At two facilities, an on-call OB/Gyn provider was used to assist with emergent cases prior to transfer to the ward. On call speciality services varied across sites.

### Clinical services

None of the facilities had clinical protocols (e.g. those for managing asthma, sepsis, DKA, etc.) (Additional file 3). There were no protocols in place for communication of critical lab results, patient or staff safety, or emergency response. Additionally, there were no protocols for infection control measures such as isolation of infectious patients or management of hazardous waste. Emergency care areas had some safety features in place, but most were not maintained. Protocols for flow through emergency care areas, including triage, patient disposition, and communication, did not exist. Two emergency care areas had dedicated spaces for triage, and all were able to obtain vital signs on patients on arrival. No formal triage systems exist.

### Signal functions

All hospitals were able to assess vital signs in emergency care areas, but the two regional hospitals were often not able to obtain pulse oximetry due to lack of equipment (Additional file 4).

For airway interventions, all hospitals were able to administer oxygen and bronchodilator therapy when equipment was functional and available. Only the tertiary hospital could always perform manual manoeuvres to open an airway as well as bag-valve-mask ventilation; regional hospitals were often limited in performing these functions due to lack of equipment and training. More advanced airway procedures such as nasopharyngeal/oropharyngeal airways, supraglottic airway device placement, and endotracheal intubations, as well as invasive and non-invasive mechanical ventilation, were rarely performed in any facility due to lack of training and equipment. Most providers in all three hospitals had the training and skills needed to perform needle thoracostomies or place chest tubes; however, absent equipment was often a barrier to provision.

Circulatory emergencies were almost always able to be managed on a basic level at all facilities. Providers in all hospitals were consistently able to administer oral rehydration, establish intravenous (IV) access, and administer IV fluids, though all faced challenges in adjusting IV fluids for cases of malnutrition or severe anaemia. The tertiary hospital and one regional hospital were sometimes able to obtain central venous access; none could provide intraosseous access or venous cutdown due to a lack of training. Pelvic binders were not used in any emergency care areas due to absent equipment and provider knowledge; these same barriers also limited the provision of safe blood transfusions. Thrombolytics were only available at the tertiary hospital. Electrocardiograms and point-of-care ultrasound were often unavailable due to absent equipment and lack of training for interpretation. For the same reasons, external defibrillation, cardioversion, pericardiocentesis, and external cardiac pacing were generally not available in any of the facilities.

All emergency care areas could assess patients using a mental status examination and perform basic neurologic interventions such as checking and managing blood glucose levels, administering benzodiazepines, managing extreme temperatures and providing physical restraint. Lumbar punctures were performed regularly at the tertiary emergency care area and one regional hospital emergency care area: at the other, they were limited by trained provider availability. Procedural sedation was not typically performed in emergency care areas and instead was reserved for the ward or operating theatre. Locally appropriate antidotes were rarely administered at any facility due to stockouts.

IV antibiotics for sepsis were usually available in all emergency care areas; two could administer IV vasopressors. The tertiary hospital was able to perform diagnostic paracentesis and all three facilities could perform minor surgical techniques for source control.

Traumatic injuries, which were seen exclusively in casualty units, were also managed well at a basic level. Providers in all emergency care areas were generally able to perform initial wound care and immobilisation of fractures, with most facilities being able to perform closed reductions of fractures and dislocations as well as immobilising the cervical spine. Antibiotics and opioids were always administered for open fractures when in stock. Staff in all emergency care areas were able to place urinary catheters, perform external haemorrhage control, and perform bleeding control with tourniquets. Packing and suturing to control bleeding could be done in two facilities and was only limited in the third due to stock outs. Adrenaline could be administered at all hospitals. None of the facilities were able to apply three-way dressings for sucking chest wounds or perform fasciotomies or escharotomies in emergency care areas: these procedures were typically performed in the operating theatre.

All hospitals were able to perform assisted vaginal deliveries in emergency care areas, if needed. Uterotonic drugs were unavailable, and neonatal resuscitation efforts were limited due to equipment, training, and personnel.

## Discussion

Emergency care was first assessed in Eswatini in 2014. Surveys identified that emergency conditions were placing severe strain on most hospitals: the combination of inadequate equipment and staffing along with high patient volumes led to reports of running over capacity at three-quarters of all facilities [10]. The HEAT showed that substantial challenges remain across referral hospitals. It highlighted strengths and weaknesses of emergency care delivery within each emergency care area and identified specific causes of these system and service gaps. The assessment process identified in each emergency care area that will allow planners to develop a targeted strategy and change management plans to effect improvements. It is likely that periodic assessments will be of use in monitoring internal improvement efforts and changes in emergency care capacity over time.

Approximately one in five patients accessed these emergency care areas via ambulance: many critical patients were transferred via ambulance. Despite estimates that admission rates did not differ greatly between the regional and tertiary hospitals, the tertiary hospital utilised substantially more inpatient beds throughout the year; this may be reflective of the longer hospital stays that more complex patients often require as they go up the referral chain [13].

All sites provided some level of emergency care 24 h a day, 7 days a week. This is fundamental to ensure that care is not delayed for critically ill and injured patients. However, casualty units and OPDs were always separated and, at two hospitals, these sites were situated across campus from one another. This is less than ideal in comparison to an emergency unit, which can provide adequate care for all patients at all times in a single space. Integration of casualty and OPD spaces is idealistic and may not be immediately feasible. In the short-term, other improvements, such as cross-training and increased communication between OPD and casualty units, could improve the situation.

The adequacy of other infrastructure aligned with previous assessments [10]. Similar to most LMIC settings, facilities struggled to provide adequate resuscitation care [14]. Regional hospitals did not have dedicated space for resuscitation, and participants at all sites noted minimal training on the subject. Although two of three hospitals (the tertiary facility, and one regional) had dedicated triage areas within their emergency care areas, none had triage protocols or training. These outcomes are consistent with other LMICs, including Sierra Leone [3, 15] and Malawi [16]. Improvement in signal function performance has consistently been linked with better outcomes across a range of settings [12, 17–19].

### Limitations

Strengthening emergency care starting at the level of referral hospitals was an initial priority of the MOH. Because of this, only regional and tertiary hospitals were considered for inclusion. Generalisability of survey results is limited in that this study included a small sample representative only of higher-level hospitals. Exhaustive sampling, where possible, may resolve such an issue but is not necessary for hospitals utilising the tool as an internal evaluation to identify emergency care gaps. It would be valuable in a future study to use the same assessment tool at first-level hospitals in order to provide a more generalisable assessment of emergency care.

Because many of the results are self-reported, they are dependent on the knowledge of particular respondents. To mitigate this, multiple respondents were interviewed independently, and average values were reported.

The HEAT is also limited in that, as a recently developed tool, it is not yet validated. However, it is based on components of other tools that are broadly validated and documented to be effective in the African setting.

## Conclusions

In order to improve emergency care and outcomes in Eswatini, additional training is likely needed relating to triage and resuscitation, as well as trauma and airway interventions, and neonatal care. Given the time-sensitive nature of emergencies, it is crucial that all facilities – regardless of their place within the referral chain – are able to care for emergencies that present to their respective emergency care areas. While this study did not look at lower-level hospitals, studies in Zambia and Tanzania found that capacity for emergency care decreases down the referral chain ^24 25^. Therefore, it is likely that emergency care system and training interventions will be most effective at mid- and low-level hospitals, where patients are likely to first present.

The lack of protocols for both patient care and administrative duties, in addition to some providers lacking the skills to provide some basic emergency care functions, suggests that all facilities could benefit from standardised trainings on both the administrative and clinical levels, such as the WHO Basic Emergency Care Course and the WHO Emergency Unit Management Course.

## Supplementary information

**Additional file 1.** Appendix 1: Eswatini EU facility characteristics

**Additional file 2.** Appendix 2: Human resources available to Eswatini EUs

**Additional file 3.** Appendix 3: Clinical services available to Eswatini EUs

**Additional file 4.** Appendix 4: Signal function performance in Eswatini EUs

## Data Availability

The datasets supporting the conclusions of this article are included within the article and its additional files. The HEAT tool can be made available upon reasonable request to WHO.

## Abbreviations

HEAT: Hospital emergency unit assessment tool
IV: Intravenous
LMICs: Low- and middle-income countries
MoH: Ministry of health
Ob/Gyn: Obstetrics and gynaecology
OPD: Outpatient department
