# Supplementary material for: Evaluating capacity at three government referral hospital emergency units in the kingdom of Eswatini using the WHO Hospital Emergency Unit Assessment Tool
Source: BMC Emerg Med. 2020 May 6;20:33. doi: 10.1186/s12873-020-00327-w (PMC7201969; doi:10.1186/s12873-020-00327-w)
Supplement: Supplementary file 1 — Additional file 1. Appendix 1: Eswatini EU facility characteristics [file 12873_2020_327_MOESM1_ESM.docx]

**Appendix 1: Eswatini EU facility characteristics.**

| **Facility characteristics** | **Regional hospital rating (median)*** | **Identified barrier(s)**** | **Tertiary hospital rating (median)*** | **Identified barrier(s)**** |
| --- | --- | --- | --- | --- |
| **Infrastructure and essential equipment** | | | | |
| Clean, running water | 2.67 | 1, 3 | 2.5 | 1 |
| Electricity source (e.g. wired, generator) | 2.67 | 1 | 3 | -- |
| Designated telephone/radio for communicating with other facilities/prehospital providers | 3 | -- | 3 | -- |
| Paper-based EU chart | 2.5 | 4 | 3 | -- |
| Electronic EU chart | 1 | 1, 5 | 1 | 1 |
| Isolation room for infectious disease (e.g. TB, haemorrhagic fever) | 1.33 | 1 | 2 | 1 |
| Easy physical access to EU for those in wheelchair or stretcher | 2.83 | 1 | 3 | -- |
| Designated waiting area | 2.17 | 1, 2 | 2.5 | 1 |
| Designated triage area | 2.17 | 1, 5, 6 | 3 | -- |
| Designated resuscitation area | 1.17 | 1, 2, 6 | 3 | -- |
| Personal protective equipment | 2.33 | 2, 4, 5, 6 | 2 | 4 |
| Electronic cardiac monitoring in EU | 1.33 | 2, 5, 6 | 1.5 | 2, 3, 5 |
| Crash trolley or code card with high-acuity equipment/supplies | 1.67 | 2, 3, 4, 6 | 2.5 | 4 |
| Rapid access to a transport ambulance and provider | 1.83 | 1, 2, 3, 6, 8 | 1.5 | 1, 2, 3, 4, 6, 7, 8 |
| Dedicated mechanism for communication with other facilities | 3 | 1, 4 | 2 | 1 |
| Is there access to storage space within the EU? | 1.83 | 1, 2 | 2 | 1 |
| Access to dedicated staff work area | 2.17 | 1, 4 | 2.5 | 1 |
| Access to toilet facilities for patients and staff | 3 | -- | 2.5 | 1 |
| Access to handwashing facilities in each patient care area | 2.33 | 1, 4 | 3 | -- |
| System for stocking, managing and dispensing medications | 2.33 | 1, 4 | 1.5 | 1, 4, 8 |
| Oxygen in EU | 2.33 | 2, 3, 4 | 2.5 | 4 |
| **Diagnostic services** | | | | |
| Haemoglobin | 3 | -- | 3 | -- |
| Full blood count | 3 | -- | 3 | -- |
| Coagulation profile (PT/PTT) | 2.5 | 2, 3, 4, 6 | 3 | -- |
| Electrolytes | 2.67 | 4 | 3 | -- |
| BUN and creatinine | 2.25 | 4 | 3 | -- |
| Lipase | 1.5 | 4 | 2.5 | 3, 4 |
| Cardiac marker (e.g. troponin) | 2 | 4 | 3 | -- |
| Arterial blood gas | 1.5 | 2, 4, 5 | 3 | -- |
| Cross matching for blood and blood products | 2.33 | 4 | 2 | 4 |
| Blood cultures | 2 | 1, 2, 3, 4, 5, 6, 9, 10 | 2 | 2, 4, 5 |
| Capacity to obtain sterile blood samples for lab testing | 2.83 | 5, 6 | 3 | -- |
| System for reporting lab results in a timely fashion | 2.67 | 1, 4, 5, 6 | 2 | 1, 2, 6 |
| Urine dipstick | 1 | 1, 2, 5, 6 | 1 | 1, 2, 5, 6 |
| Urine pregnancy | 1.67 | 1, 2, 4, 5, 8 | 1.5 | 1, 2, 4, 5, 6 |
| Glucose | 2.5 | 2, 4 | 3 | -- |
| Malaria Rapid Diagnostic Test (RDT) | 1.17 | 1, 2, 4, 5, 6 | 2 | 1, 2, 5, 6 |
| Rapid HIV testing | 2 | 1, 4, 6, 8 | 2 | 6, 8 |
| Stationary X-ray | 2.5 | 1, 2 | 3 | -- |
| Portable X-ray for use in EU | 1 | 1, 2 | 1 | 2 |
| Ultrasound for use in hospital | 1.67 | 5, 6, 8 | 3 | -- |
| Ultrasound for use in EU | 1 | 2, 5, 6 | 1 | 2 |
| CT scan | 1 | 1, 2, 5, 6, 9 | 3 | -- |
| System for reporting radiology results in a timely fashion | 2.17 | 4, 5, 6, 8, 9 | 1.5 | 5, 6, 9 |

*Median availability ratings across all participants at site(s), where resource, service or function was noted as: 1 - generally unavailable; 2 - somewhat available (available to only some of those who need it); or 3 - adequate (present and available to almost everyone in need and used when needed).

** Barriers to availability of critical HEAT resources, services, and functions are described in Table 1.
