# Supplementary material for: Evaluating capacity at three government referral hospital emergency units in the kingdom of Eswatini using the WHO Hospital Emergency Unit Assessment Tool
Source: BMC Emerg Med. 2020 May 6;20:33. doi: 10.1186/s12873-020-00327-w (PMC7201969; doi:10.1186/s12873-020-00327-w)
Supplement: Supplementary file 2 — Additional file 2. Appendix 2: Human resources available to Eswatini EUs [file 12873_2020_327_MOESM2_ESM.docx]

**Appendix 2: Human resources available to Eswatini EUs.**

| **Human Resources** | **Regional hospital rating (median)*** | **Identified barrier(s)**** | **Tertiary hospital rating (median)*** | **Identified barrier(s)**** |
| --- | --- | --- | --- | --- |
| **Consulting services available to the EU** | | | | |
| General surgery | 2.6 | 6, 8 | 3 | -- |
| Obstetrics and gynaecology | 2.6 | 6, 8 | 3 | -- |
| Orthopaedics | 1.8 | 5, 6, 8 | 3 | -- |
| Anaesthesia | 2.8 | 6, 8 | 3 | -- |
| Paediatrics | 2.6 | 6, 8 | 3 | -- |
| Psychiatry | 1.4 | 5, 6, 8 | 1 | 5, 6 |
| Other consulting services available | 2.5 | ENT  PT  Dental  Maxillofacial | 3 | Dental  Cardiology  Ophthalmology  ENT  GI  Neurosurgery |

*Median availability ratings across all participants at site(s), where resource, service or function was noted as: 1 - generally unavailable; 2 - somewhat available (available to only some of those who need it); or 3 - adequate (present and available to almost everyone in need and used when needed).

** Barriers to availability of critical HEAT resources, services, and functions are described in Table 1.
