# Supplementary material for: Evaluating capacity at three government referral hospital emergency units in the kingdom of Eswatini using the WHO Hospital Emergency Unit Assessment Tool
Source: BMC Emerg Med. 2020 May 6;20:33. doi: 10.1186/s12873-020-00327-w (PMC7201969; doi:10.1186/s12873-020-00327-w)
Supplement: Supplementary file 3 — Additional file 3. Appendix 3: Clinical services available to Eswatini EUs [file 12873_2020_327_MOESM3_ESM.docx]

**Appendix 3: Clinical services available to Eswatini EUs.**

| **Clinical Services** | **Regional hospital rating (median)*** | **Identified barrier(s)**** | **Tertiary hospital rating (median)*** | **Identified barrier(s)**** | **Average Overall Hospital Rating** |
| --- | --- | --- | --- | --- | --- |
| **Ancillary services** | | | | | |
| Social work services | 1.63 | 5, 6, 8 | 1.5 | 5, 6, 8 | 1.57 |
| Patient transport services | 1.63 | 2, 6 | 1.5 | 2, 3, 6 | 1.57 |
| Security | 2.38 | 5, 6, 8 | 2.5 | 6 | 2.75 |

*Median availability ratings across all participants at site(s), where resource, service or function was noted as: 1 - generally unavailable; 2 - somewhat available (available to only some of those who need it); or 3 - adequate (present and available to almost everyone in need and used when needed).

** Barriers to availability of critical HEAT resources, services, and functions are described in Table 1.
