# Supplementary material for: Evaluating capacity at three government referral hospital emergency units in the kingdom of Eswatini using the WHO Hospital Emergency Unit Assessment Tool
Source: BMC Emerg Med. 2020 May 6;20:33. doi: 10.1186/s12873-020-00327-w (PMC7201969; doi:10.1186/s12873-020-00327-w)
Supplement: Supplementary file 4 — Additional file 4. Appendix 4: Signal function performance in Eswatini EUs [file 12873_2020_327_MOESM4_ESM.docx]

**Appendix 4: Signal function performance in Eswatini EUs.**

| **Signal function performance** | **Regional hospital rating (median)*** | **Identified barrier(s)**** | **Tertiary hospital rating (median)*** | **Identified barrier(s)**** |
| --- | --- | --- | --- | --- |
| **Vitals signs** | | | | |
| Are vital signs measured in the triage area? | 2.67 | 1 | 3 | -- |
| Are vital signs measured in the EU? | 3 | -- | 3 | -- |
| **Airway interventions** | | | | |
| Use of manual manoeuvres | 1.67 | 5, 6 | 3 | -- |
| Use of suction | 2.4 | 3, 4, 5, 6 | 2.5 | 3, 5 |
| Placement of oro- or nasopharyngeal airway device | 1.6 | 2, 5, 6 | 1.5 | 2, 5 |
| Placement of supraglottic device (e.g. LMA | 1.8 | 2, 5, 6 | 1 | 2, 5 |
| Endotracheal intubation | 2 | 2, 5, 6 | 1.5 | 2, 5, 6 |
| Creation of surgical airway | 1.8 | 1, 2, 5, 6 | 1 | 1, 2, 5, 6 |
| **Breathing interventions** | | | | |
| Measurement of pulse oximetry at triage | 2.5 | 3 | 3 | -- |
| Measurement of pulse oximetry in EU treatment areas | 2.33 | 2, 3 | 2 | 2 |
| Administration of bronchodilator for reactive airway disease | 2.33 | 4 | 3 | -- |
| Administration of oxygen | 2.67 | 2, 3, 4 | 3 | -- |
| Bag-valve-mask ventilation | 2.17 | 4, 5 | 3 | -- |
| Non-invasive mechanical ventilation | 1.5 | 2, 4, 5 | 2 | 2, 5 |
| Invasive mechanical ventilation | 1.17 | 1, 2, 5, 6 | 1 | 1, 2, 5 |
| Perform needle decompression of tension pneumothorax | 2.33 | 3, 4, 5, 6 | 3 | -- |
| Placement of chest tube | 2.5 | 3, 4, 6 | 3 | -- |
| **Circulation interventions** | | | | |
| Administer oral rehydration | 2.83 | 4 | 3 | -- |
| Place peripheral IV | 2.83 | 4 | 3 | -- |
| Establish IO access | 1.5 | 2, 4, 5, 6 | 1 | 2, 5 |
| Perform venous cutdown | 1.6 | 2, 4, 5, 6 | 1 | 5 |
| Establish central venous access | 2 | 2, 4, 5, 6, 8 | 2.5 | 5, 6 |
| Administration of IV fluids | 2.67 | 4 | 3 | -- |
| Adjust fluids for malnutrition/severe anaemia | 2.17 | 4, 5 | 2 | 5 |
| Place urinary catheter | 2.5 | 4 | 3 | -- |
| External control of haemorrhage | 2.83 | 5 | 3 | -- |
| Perform packing and/or suture control | 2.83 | 4, 6 | 3 | -- |
| Apply arterial tourniquet | 2.83 | 4, 6 | 3 | -- |
| Apply pelvic binding or sheeting | 1.33 | 2, 4, 5 | 1 | 2, 5 |
| Ability to perform safe transfusion | 2 | 1, 2, 4, 5, 6, 9 | 1.5 | 2, 3, 4, 5 |
| Perform and interpret POC ultrasound | 1 | 1, 2, 5, 6 | 1 | 2, 5 |
| Perform pericardiocentesis | 1 | 1, 2, 5, 6 | 2 | 9 |
| Perform external defibrillation and/or cardioversion | 1.33 | 2, 5 | 1.5 | 1, 2, 5 |
| Perform external cardiac pacing | 1 | 2, 5, 6 | 1.5 | 1, 2, 5 |
| Administration of adrenaline | 2.83 | 4 | 3 | -- |
| Perform and interpret ECG | 1.5 | 2, 3, 4, 5, 8 | 1.5 | 3, 5 |
| Administer aspirin for ischemia | 2.17 | 4, 5, 9 | 3 | -- |
| Administration of thrombolytics for MI | 1.33 | 2, 4, 5 | 1.5 | 1, 4, 5 |
| **Neurologic interventions** | | | | |
| Check glucose level | 2.67 | 4 | 3 | -- |
| Administer glucose for hypoglycaemia | 2.83 | 4 | 3 | -- |
| Administer insulin for hyperglycaemia | 2.83 | 4 | 3 | -- |
| Perform lumbar puncture | 2.83 | 6 | 2 | 2, 5 |
| Protect from secondary injury | 2.17 | 1, 2, 5, 6 | 2.5 | 1, 2 |
| Administer benzodiazepines | 2.5 | 4, 5 | 3 | -- |
| Administer IV magnesium for pregnant patients | 2 | 1, 4, 5, 6 | 1 | 4, 5 |
| Administer locally appropriate antidote | 1.5 | 4, 5 | 1.5 | 4, 5 |
| Perform mental status exam | 3 | -- | 3 | -- |
| Management of extreme temperatures | 2.83 | 4 | 3 | -- |
| Ability to provide physical restraints | 2.17 | 2, 4, 5, 6, 9 | 3 | -- |
| Administer appropriate therapeutics for agitation | 2.17 | 4, 5, 6, 8 | 1 | 4, 5, 9 |
| Perform procedural sedation | 2.5 | 4 | 2.5 | 1 |
| **Sepsis interventions** | | | | |
| Administration of IV antibiotics | 2.67 | 4 | 2.5 | 4 |
| Administration of IV vasopressors | 2.17 | 4 | 2 | 4, 5 |
| Perform diagnostic paracentesis | 2.5 | 5, 6 | 2 | 2, 5 |
| Bedside minor surgical techniques for source control | 2.83 | 2, 3, 4, 5 | 2 | 1 |
| **Trauma interventions** | | | | |
| Immobilise the cervical spine | 1.67 | 2, 4, 5 | 2.5 | 2, 3 |
| Apply three-way dressing for sucking chest wound | 1.5 | 2, 4, 5 | 1 | 1, 5 |
| Perform fasciotomy or escharotomy | 1.6 | 1, 5, 6 | 1 | 1, 2, 5 |
| Administer opiate analgesia | 2.33 | 4, 9 | 2.5 | 4 |
| Immobilise fractures | 3 | -- | 3 | -- |
| Perform closed reduction of fracture or dislocation | 2.5 | 2, 5, 6 | 2 | 1, 5 |
| Administer antibiotics for open fracture | 2.67 | 4 | 3 | -- |
| Perform appropriate initial wound care | 2.67 | 2, 4 | 3 | -- |
| Administer tetanus vaccination or IVIG | 2 | 2, 4 | 1.5 | 1, 9 |
| Administer rabies vaccine or IVIG | 2.83 | 4 | 2 | -- |
| **Obstetric interventions** | | | | |
| Perform assisted vaginal delivery | 2.17 | 2, 5, 6, 8 | 2.5 | 1, 2, 5, 6 |
| Administer uterotonic drug | 1.83 | 2, 4, 5, 9 | 1 | 4, 5, 9 |
| Perform neonatal resuscitation | 2.33 | 2, 4, 5, 6 | 1.5 | 2, 5 |

*Median availability ratings across all participants at site(s), where resource, service or function was noted as: 1 - generally unavailable; 2 - somewhat available (available to only some of those who need it); or 3 - adequate (present and available to almost everyone in need and used when needed).

** Barriers to availability of critical HEAT resources, services, and functions are described in Table 1.
